# SLMSF-Net: A Semantic Localization and Multi-Scale Fusion Network for RGB-D Salient Object Detection

**DOI:** 10.3390/s24041117

**Published:** 2024-02-08

**Authors:** Yanbin Peng, Zhinian Zhai, Mingkun Feng

**Affiliations:** School of Information and Electronic Engineering, Zhejiang University of Science and Technology, Hangzhou 310023, China

**Keywords:** RGB-D, salient object detection, multi-modal and multi-scale features

## Abstract

Salient Object Detection (SOD) in RGB-D images plays a crucial role in the field of computer vision, with its central aim being to identify and segment the most visually striking objects within a scene. However, optimizing the fusion of multi-modal and multi-scale features to enhance detection performance remains a challenge. To address this issue, we propose a network model based on semantic localization and multi-scale fusion (SLMSF-Net), specifically designed for RGB-D SOD. Firstly, we designed a Deep Attention Module (DAM), which extracts valuable depth feature information from both channel and spatial perspectives and efficiently merges it with RGB features. Subsequently, a Semantic Localization Module (SLM) is introduced to enhance the top-level modality fusion features, enabling the precise localization of salient objects. Finally, a Multi-Scale Fusion Module (MSF) is employed to perform inverse decoding on the modality fusion features, thus restoring the detailed information of the objects and generating high-precision saliency maps. Our approach has been validated across six RGB-D salient object detection datasets. The experimental results indicate an improvement of 0.20~1.80%, 0.09~1.46%, 0.19~1.05%, and 0.0002~0.0062, respectively in maxF, maxE, S, and MAE metrics, compared to the best competing methods (AFNet, DCMF, and C2DFNet).

## 1. Introduction

Salient Object Detection (SOD) plays a crucial role in the field of computer vision, with its primary objective being the identification and accentuation of the most visually engaging objects within a scene [1,2]. These objects typically draw the majority of observer attention and play a vital role in image and video processing tasks, such as object tracking [3,4], image segmentation [5,6], and scene understanding [7,8]. With the rapid advancement of depth sensor technology, RGB-D salient object detection has elicited significant interest among researchers. Compared to using only RGB images, RGB-D datasets offer a richer array of information, including color and depth details, which are invaluable in enhancing the performance of salient object detection. However, the achievement of accurate salient object detection under complex scenarios, with multi-scale objects and noise interference, continues to present a substantial challenge. Current research is confronted with two main issues [9,10,11,12,13,14,15,16,17,18,19,20,21,22,23,24,25,26,27,28,29,30,31,32,33,34,35,36,37]:

1. The Modality Fusion Problem: Undoubtedly, depth information opens up significant possibilities for enhancing detection performance. The distance information it provides between objects aids in clearly distinguishing the foreground from the background, thereby endowing the algorithm with robustness when dealing with complex scenarios. However, an urgent challenge that remains to be solved is how to fully exploit this depth information and effectively integrate it with the color, texture, and other features of RGB images to extract richer and more discriminative features. This challenge becomes particularly pressing when dealing with issues of incomplete depth information and noise interference, which necessitate further exploration and research.
2. The Multi-level Feature Integration Problem: To more effectively integrate multi-level features, it’s vital to fully consider the characteristics of both high-level and low-level features. High-level features contain discriminative semantic information, which aids in the localization of salient objects, while low-level features are rich in detailed information, beneficial for optimizing object edges. Traditional RGB-D salient object detection methods often fuse features from different levels directly, disregarding their inherent differences. This approach can lead to semantic information loss and make the method vulnerable to noise and background interference. Therefore, there is a need to explore more refined feature fusion techniques that fully take into account the characteristics of different levels of features, aiming to boost the performance of salient object detection.

To address the aforementioned challenges, we propose a Semantic Localization and Multi-Scale Fusion Network (SLMSF-Net) for RGB-D salient object detection. SLMSF-Net constitutes two stages: encoding and decoding. During the encoding phase, SLMSF-Net utilizes the ResNet50 network to separately extract features from RGB and depth images and employs a depth attention module for modal feature fusion. In the decoding phase, SLMSF-Net first accurately localizes salient objects through a semantic localization module, and then constructs a reverse decoder using a Multi-Scale Fusion Module to restore the detailed information of the salient objects. Our main contributions can be summarized as follows:

1. We propose a depth attention module that leverages channel and spatial attention mechanisms to fully explore the effective information of depth images and enhance the matching ability between RGB and depth feature maps.
2. We propose a semantic localization module that constructs a global view for the precise localization of salient objects.
3. We propose a reverse decoding network based on multi-scale fusion, which implements reverse decoding on modal fusion features and generates detailed information on salient objects through multi-scale feature fusion.

The design of the SLMSF-Net is poised to address key issues in the current RGB-D salient object detection domain and provide new research insights for other tasks within the field of computer vision. Extensive experimental results fully demonstrate that the SLMSF-Net exhibits excellent performance in RGB-D SOD tasks, enhancing the accuracy and effectiveness of salient object detection.

## 2. Related Works

In this section, we will review research works [17,18,19,20,21,22,23,24,25,26,27,28,29,30,31,32,33,34,35,36,37] related to the RGB-D salient object detection method that we propose. These related studies can be broadly divided into two categories: salient object detection based on RGB images and salient object detection based on RGB-D images.

### 2.1. Salient Object Detection Based on RGB Images

Salient object detection based on RGB images mainly focuses on visual cues such as color, texture, and contrast. Early saliency detection methods primarily depended on handcrafted features and heuristic rules. For instance, Itti et al. [17] proposed a saliency detection model based on the biological visual system, which estimates saliency by calculating the local contrast of color, brightness, and directional features. Achanta et al. [18] introduced a frequency-tuned salient region detection method, which extracts global contrast features in the frequency domain of the image to detect salient regions. Tong et al. [19] combined global and local cues for salient object detection, using a variety of cues (such as color, texture, and contrast) to handle complex scenarios.

In recent years, deep learning technology has achieved significant success in the field of salient object detection. Models such as the deep learning saliency model proposed by Chen et al. [20] accomplish hierarchical representation of saliency features to realize end-to-end salient object detection. Cong et al. [21] proposed a salient object detection method based on a Fully Convolutional Network (FCN), which uses global contextual information and local detail information for saliency prediction. Hou et al. [22] developed a deeply supervised network for salient object detection, improving upon the Holistically Nested Edge Detector (HED) architecture. They introduced short connections between network layers, enhancing salient object detection by combining low-level and high-level features. Zhao et al. [23] proposed GateNet, a new network architecture for salient object detection. This model introduced multilevel gate units to balance encoder block contributions, suppressing non-salient features and contextualizing for the decoder. They also included Fold-ASPP to gather multiscale semantic information, enhancing atrous convolution for better feature extraction. Zhang et al. [24] combined neural network layer features to improve salient object detection accuracy in images. Their approach used both coarse and fine image details and incorporated edge-aware maps to enhance boundary detection. Wu et al. [25] proposed a cascaded partial decoder that discarded low-level features to reduce computational complexity while refining high-level features for accuracy.

Moreover, some researchers have applied attention mechanisms to RGB-based salient object detection models, such as [26,27,28]. These methods enable the models to concentrate their attention on the visually prominent regions of the image. Chen et al. [26] presented an approach for enhancing salient object detection through the use of reverse attention and side-output residual learning. This method aimed to refine saliency maps with a particular focus on improving resolution and reducing the model’s size. Wang et al. [27] presented PAGE-Net, a model for salient object detection. The model utilized a pyramid attention module to enhance saliency representation by incorporating multi-scale information, thereby effectively boosting detection accuracy. Additionally, it featured a salient edge detection module, which sharpened the detection of salient object boundaries. Wang et al. [28] introduced PiNet, a salient object detection model designed for enhancing feature extraction and the progressive refinement of saliency. The model incorporated level-specific feature extraction mechanisms and employed a coarse-to-fine process for refining saliency features, which helped in overcoming common issues in existing methods like noise accumulation and spatial detail dilution. Although methods based on RGB images can achieve good performance in many situations, they lack the ability to handle depth information.

### 2.2. Salient Object Detection Based on RGB-D Images

With the advancement of depth sensors, RGB-D images (which contain both color and depth information) have been widely applied in salient object detection. For instance, Lang et al. [29] investigated the impact of depth cues on saliency detection, where they found that depth information holds significant value for salient object detection. Based on this, many researchers have begun to explore how to fully utilize depth information for salient object detection.

Peng et al. [30] proposed a multi-modal fusion framework that improves saliency detection performance by fusing local and global depth features with color and texture features. Zhang et al. [31] presented a new RGB-D salient object detection model, addressing challenges with depth image quality and foreground–background consistency. The model introduced a two-stage approach: firstly, an image generation stage that created high-quality, foreground-consistent pseudo-depth images, and secondly, a saliency reasoning stage that utilized these images for enhanced depth feature calibration and cross-modal fusion. Ikeda et al. [32] introduced a model for RGB-D salient object detection that integrated saliency and edge features with reverse attention. This approach effectively enhanced object boundary detection and saliency in complex scenes. The model also incorporated a Multi-Scale Interactive Module for improved global image information understanding and utilized supervised learning to enhance accuracy in salient object and boundary areas. Xu et al. [33] introduced a new approach to RGB-D salient object detection, addressing the object-part relationship dilemma in Salient Object Detection (SOD). The proposed CCNet model utilized a Convolutional Capsule Network based on Feature Extraction and Integration (CCNet) to efficiently explore the object-part relationship in RGB-D SOD with reduced computational demand. Cong et al. [34] presented a comprehensive approach to RGB-D salient object detection, focusing on enhancing the interaction and integration of features from both RGB and depth modalities. It introduced a new network architecture that efficiently combined these modalities, addressing challenges in feature representation and fusion. However, these methods overlook the feature differences between different modalities, resulting in insufficient information fusion.

To address this issue, Qu et al. [35] introduced a simple yet effective deep learning model, which learns the interaction mechanism between RGB and depth-induced saliency features. Yi et al. [36] proposed a Cross-stage Multi-scale Interaction Network (CMINet), which intertwines features at different stages with the use of a Multi-scale Spatial Pooling (MSP) module and a Cross-stage Pyramid Interaction (CPI) module. They then designed an Adaptive Weight Fusion (AWF) module for balancing the importance of multi-modal features and fusing them. Liu et al. [37] proposed a cross-modal edge-guided salient object detection model for RGB-D images. This model extracts edge information from cross-modal color and depth information and integrates the edge information into cross-modal color and depth features, generating a saliency map with clear boundaries. Sun et al. [38] introduced an RGB-D salient object detection method that combined cross-modal interactive fusion with global awareness. This method embedded a transformer network within a U-Net structure to merge global attention mechanisms with local convolution, aiming for enhanced feature extraction. It utilized a U-shaped structure for extracting dual-stream features from RGB and depth images, employing a multi-level information reconstruction approach to suppress lower-layer disturbances and minimize redundant details. Peng et al. [39] introduced MFCG-Net, an RGB-D salient object detection method that leveraged multimodal fusion and contour guidance to improve detection accuracy. It incorporated attention mechanisms for feature optimization and designed an interactive feature fusion module to effectively integrate RGB and depth image features. Additionally, the method utilized contour features to guide the detection process, achieving clearer boundaries for salient objects. Sun et al. [40] introduced a new approach for RGB-D salient object detection, leveraging a cascaded and aggregated Transformer Network structure to enhance feature extraction and fusion. They employed three key modules: the Attention Feature Enhancement Module (AFEM) for multi-scale semantic information, the Cross-Modal Fusion Module (CMFM) to address depth map quality issues, and the Cascaded Correction Decoder (CCD) to refine feature scale differences and suppress noise. Although some significant results have been achieved in existing research, it remains a formidable challenge to achieve accurate salient object detection in complex scenes through cross-modal and cross-level feature fusion.

## 3. Proposed Method

In this section, we first provide an overview of our method in Section 3.1. Following that, in Section 3.2, we elaborate on the depth attention module we propose, which is used to mine valuable depth information. In Section 3.3 and Section 3.4, we introduce the semantic localization module and the reverse decoding network based on the Multi-Scale Fusion Module, respectively. Finally, in Section 3.5, we discuss the loss function.

### 3.1. Overview of SLMSF-Net

**Figure 1:**
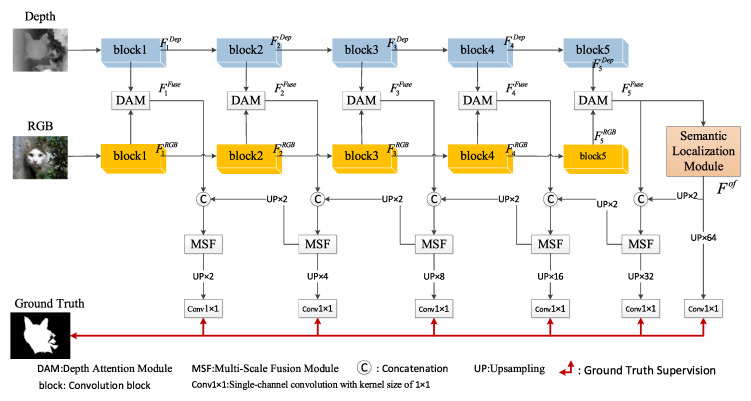
The overall network architecture of SLMSF-Net.

Figure 1 displays the overall network structure of SLMSF-Net. Without loss of generality, we adopt Resnet50 [41] as the backbone network to extract features from both RGB images and depth images separately. Resnet50 encompasses five convolution stages; we removed the final pooling layer and the fully connected layer, resulting in a fully convolutional neural network, and use the outputs of the intermediate five convolution blocks as feature outputs. These output feature maps are denoted as M1, M2, M3, M4, and M5, with their sizes being 1/2, 1/4, 1/8, 1/16, and 1/32 of the original image, respectively.

1. Modal Feature Fusion: As shown in Figure 1, we proposed a Depth Attention Module. This module performs a modal fusion of RGB image features and depth image features, forming the modal fusion features $F_{1}^{Fuse}$, $F_{2}^{Fuse}$, $F_{3}^{Fuse}$, $F_{4}^{Fuse}$ and $F_{5}^{Fuse}$.
2. Semantic Localization: We proposed a Semantic Localization Module. This module first downsamples the top-level modal fusion feature to compute a global view. It then performs coordinate localization on the global view and ultimately fuses the localization information with the global view, thereby precisely locating the salient object. Assuming the semantic localization module is represented as the SLM function, its output result can be written as: $F^{of}=\mathrm{SLM}(F_{5}^{Fuse})$.
3. Multi-Scale Fusion Decoding: After performing semantic localization, we predicted the clear boundaries of the salient object through reverse multi-level feature integration from front to back. To accomplish this multi-level feature integration, we constructed a Multi-Scale Fusion Module, which effectively fuses features at all levels.

### 3.2. Depth Attention Module

In the process of fusing RGB and depth features, we need to address two main issues. The first one is the modal mismatch problem, which requires us to resolve the modal differences between the two types of features. The second one is the information complementarity problem; since RGB and depth features often capture different aspects of object information, we need to consider how to let these two types of features complement each other’s information, aiming to enhance the accuracy and robustness of object detection. Inspired by [42], we designed a depth attention module to improve the matching and complementarity of multi-modal features.

**Figure 2:**
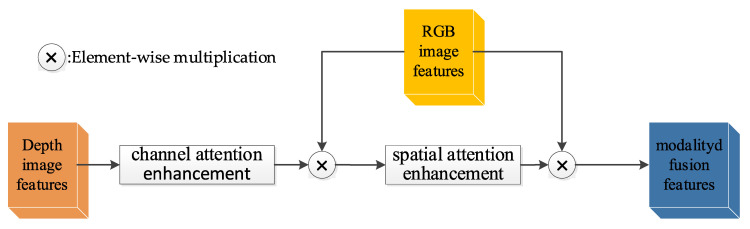
Depth attention module.

Specifically, $F_{i}^{RGB}$ represents the ith RGB image feature and $F_{i}^{Dep}$ represents the *i*th depth image feature, where *i* is a natural number from 1 to 5. As shown in Figure 2, the depth attention module first enhances the depth image feature through channel attention. The enhanced result is then multiplied element-wise with the RGB image feature to obtain the channel-enhanced fusion feature. Following this, the channel-enhanced fusion feature undergoes spatial attention enhancement, and the enhanced result is multiplied element-wise with the RGB image feature, thus obtaining the modal fusion feature. To enhance the matching of depth features, we stacked a depth attention module behind each depth feature branch. By introducing attention units, we can enhance the saliency representation ability of depth features. The fusion process of the two modal features can be expressed as follows:

(1) $$F_{i}^{\mathrm{Fuse}}=F_{i}^{\mathrm{RGB}}\times \mathrm{SA}(F_{i}^{\mathrm{RGB}}\times \mathrm{CA}(F_{i}^{\mathrm{Dep}}))$$

Herein, CA(⋅) symbolizes the channel attention operation, SA(⋅) indicates the spatial attention operation, and × represents the element-wise multiplication operation.

### 3.3. Semantic Localization Module

**Figure 3:**
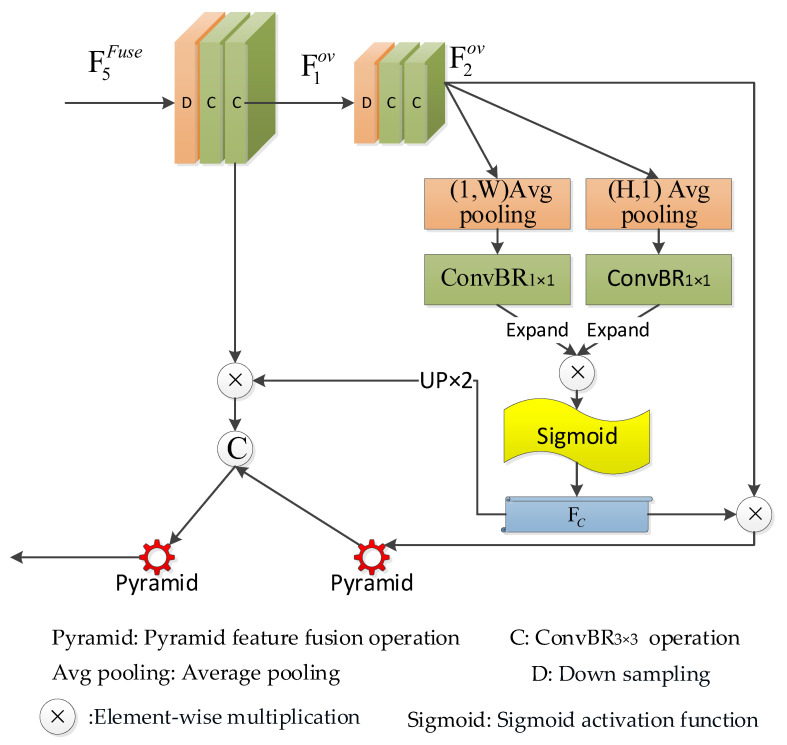
Semantic localization module.

In the process of salient object localization, high-level features play a crucial role. Compared to low-level features, high-level features are capable of capturing more abstract information, which aids in highlighting the location of salient objects. Therefore, we introduced a semantic localization module designed to effectively learn the global view of the entire image, thereby achieving more precise salient object localization. As depicted in Figure 3, the semantic localization process is divided into three stages: initially, the first stage downsamples the top-level modal fusion features to compute a global view; subsequently, the second stage carries out coordinate localization on the global view; finally, the third stage fuses the localization information with the global view.

In the first stage, we implement a 1/2 scale downsample operation on the top-level modal fusion features $F_{5}^{\mathrm{Fuse}}$, followed by two ${\mathrm{ConvBR}}_{3\times 3}$ operations, thereby obtaining the first layer of the global feature map $F_{1}^{\mathrm{ov}}$. Subsequently, we perform the same 1/2 scale downsample and two ${\mathrm{ConvBR}}_{3\times 3}$ operations on the first layer of the global feature map, resulting in the second layer of the global feature map $F_{2}^{\mathrm{ov}}$. As observed, these two global feature maps possess a significantly large receptive field, enabling them to serve as the global view of the entire image. The computation process for the global view can be described as follows:

(2) $$F_{1}^{\mathrm{ov}}{=\mathrm{ConvBR}}_{3\times 3}({\mathrm{ConvBR}}_{3\times 3}({\mathrm{DownS}}_{1/2}(F_{5}^{\mathrm{Fuse}})))$$

(3) $$F_{2}^{\mathrm{ov}}{=\mathrm{ConvBR}}_{3\times 3}({\mathrm{ConvBR}}_{3\times 3}({\mathrm{DownS}}_{1/2}(F_{1}^{\mathrm{ov}})))$$

Herein, ${\mathrm{DownS}}_{1/2}(\cdot )$ denotes a 1/2 scale downsample operation on the input feature map. ${\mathrm{ConvBR}}_{3\times 3}(\cdot )$ represents a convolution operation performed on the input feature map using a kernel size of 3×3, followed by batch normalization and activation operations, where the activation function is Relu. This can be expressed as:

(4) $${\mathrm{ConvBR}}_{3\times 3}(X)=\mathrm{Relu}(\mathrm{BN}({\mathrm{Conv}}_{3\times 3}(X)))$$

Herein, Conv(⋅) symbolizes the convolution operation, BN(⋅) denotes the batch normalization operation, and Relu(⋅) represents the Relu activation function.

In the second stage, for the second layer of the global feature map $F_{2}^{\mathrm{ov}}$, we utilize a pooling kernel of size (1, W) to perform average pooling along the vertical coordinate of the feature map, followed by a convolution operation with a kernel size of 1×1, resulting in the height-oriented feature map $T_{H}$. Simultaneously, we use a pooling kernel of size (H, 1) to conduct average pooling along the horizontal coordinate of the feature map $F_{2}^{\mathrm{ov}}$, then perform a convolution operation with a kernel size of 1×1, yielding the width-oriented feature map $T_{W}$. This can be described as:

(5) $$T_{H}={\mathrm{ConvBR}}_{1\times 1}(\frac{1}{W}\sum_{0\leq i<W}F_{2}^{\mathrm{ov}}(H,i))$$

(6) $$T_{W}={\mathrm{ConvBR}}_{1\times 1}(\frac{1}{H}\sum_{0\leq j<H}F_{2}^{\mathrm{ov}}(j,W))$$

Herein, feature map $T_{H}$ extends in the width direction, while feature map $T_{W}$ expands in the height direction. The two expanded feature maps undergo pixel-wise multiplication, and then through a Sigmoid activation function, a coordinate localization feature map $F_{C}$ is formed. This can be described as:

(7) $$F_{C}=\mathrm{Sigmoid}(K(T_{H})\times K(T_{W}))$$

Herein, the K(⋅) operation refers to expanding the input feature map in the width or height direction to match the size of feature map A, while Sigmoid(⋅) signifies the Sigmoid activation function.

In the third stage, we view the localization feature map as a self-attention mechanism for calibrating the global view. Specifically, we perform a pixel-wise multiplication operation between the localization feature map $F_{C}$ and the second layer of the global feature map $F_{2}^{\mathrm{ov}}$, followed by a pyramid feature fusion operation on the multiplication results, yielding feature map $F_{*}^{\mathrm{of}}$. Subsequently, we upscale the localization feature map $F_{C}$ twice and perform a pixel-wise multiplication operation with the first layer of the global feature map $F_{1}^{ov}$. The result is stacked with $F_{*}^{\mathrm{of}}$, and then the stacked result is subjected to a pyramid feature fusion operation to finally obtain the global localization fusion feature $F^{\mathrm{of}}$. This can be described as:

(8) $$F_{*}^{\mathrm{of}}=\mathrm{Pyramid}(F_{C}\times F_{2}^{\mathrm{ov}})$$

(9) $$F^{\mathrm{of}}=\mathrm{Pyramid}(\mathrm{concat}(\mathrm{UP}(F_{C})\times F_{1}^{\mathrm{ov}},F_{*}^{\mathrm{of}}))$$

Herein, Pyramid(⋅) represents the pyramid feature fusion operation, concat(⋅) signifies the stacking operation along the channel, and UP(⋅) denotes the operation of upscaling by a factor of two.

**Figure 4:**
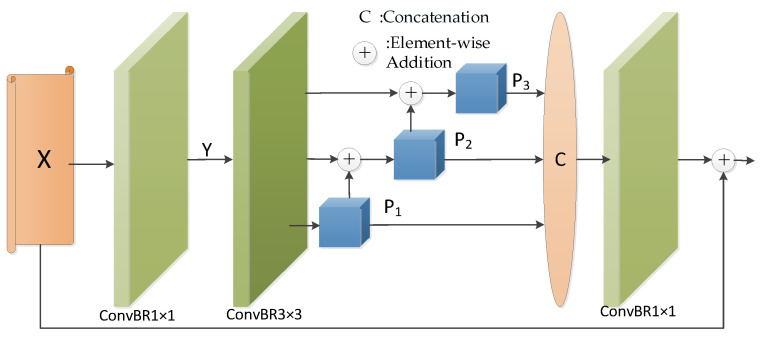
Pyramid feature fusion operation.

The pyramid feature fusion operation is depicted in Figure 4. Initially, we conduct a convolution operation with a kernel size 1×1, adjusting the number of channels in the input feature map X to 32, which yields the feature map Y. Following this, we execute feature extraction on Y, with the specific extraction method detailed as follows:

(10) $$Y{=\mathrm{ConvBR}}_{1\times 1}(X)$$

(11) $$P_{1}{=\mathrm{ConvBR}}_{3\times 3}{(\mathrm{ConvBR}}_{3\times 3}(Y))$$

(12) $$P_{i}{=\mathrm{ConvBR}}_{3\times 3}^{2i-1}{(\mathrm{ConvBR}}_{3\times 3}(Y)+P_{i-1})),\;i(i\in \{2,3\})$$

Herein, ${\mathrm{ConvBR}}_{3\times 3}^{2i-1}(\cdot )$ represents a dilated convolution with a kernel size of 3×3 and a dilation rate of 2i−1. We perform a concatenation operation along the channel with the three extracted features. Subsequently, we conduct a convolution operation on the concatenation result with a kernel size of 1×1, adjusting the channel count to match that of the input feature map. Finally, a residual connection is established with the input feature map. This process can be described as follows:

(13) $$\mathrm{Pyramid}(X)={\mathrm{ConvBR}}_{1\times 1}(\mathrm{concat}(P_{1},P_{2},P_{3},P_{4}))+X$$

### 3.4. Multi-Scale Fusion Module and the Reverse Decoding Process

Following semantic localization, we integrate multi-layer features in a forward-to-backward manner to delineate intricate details of the salient object. To achieve this multi-layer feature integration, we designed and constructed a Multi-Scale Fusion Module. The reverse decoder operates in five stages, each accepting the output from the preceding stage for reverse multi-scale fusion decoding. Importantly, the input for the fifth stage of the decoder is the global localization fusion feature $F^{\mathrm{of}}$. The process of the reverse decoder can be described as follows:

(14) $$Decode_{5}^{*}=\mathrm{UP}({\mathrm{ConvBR}}_{1\times 1}(F^{\mathrm{of}}))$$

(15) $$Decode_{5}=\mathrm{MSF}(\mathrm{concat}({\mathrm{ConvBR}}_{1\times 1}(F_{5}^{\mathrm{Fuse}}),Decode_{5}^{*}))$$

(16) $$Decode_{i}^{*}=\mathrm{UP}({\mathrm{ConvBR}}_{1\times 1}(Decode_{i+1}))$$

(17) $$Decode_{i}=\mathrm{MSF}(\mathrm{concat}({\mathrm{ConvBR}}_{1\times 1}(F_{i}^{\mathrm{Fuse}}),Decode_{i}^{*})),\;i(i\in \{1,2,3,4\})$$

Herein, MSF(⋅) stands for the Multi-Scale Fusion Module. We upscale the output from the first stage of the decoder to the size of the input image, thereby obtaining the final saliency prediction map. The specific formula used to generate the saliency prediction map is as follows:

(18) $${S=\mathrm{Sigmoid}(\mathrm{Conv}}_{1\times 1}{(\mathrm{UP}}_{\mathrm{in}}(Decode_{1})))$$

Herein, S represents the saliency prediction map, ${\mathrm{UP}}_{\mathrm{in}}(\cdot )$ denotes the upscaling of the feature map to the size of the input image, while ${\mathrm{Conv}}_{1\times 1}(\cdot )$ signifies a single-channel convolution with a kernel size of 1×1. The primary purpose of ${\mathrm{Conv}}_{1\times 1}(\cdot )$ is to adjust the channel count of the feature map to 1.

**Figure 5:**
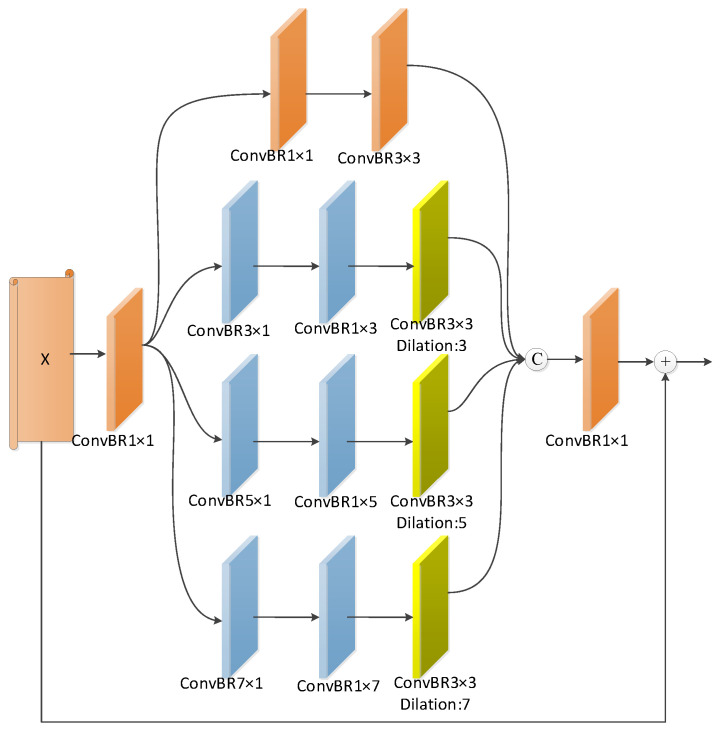
Multi-Scale Fusion Module.

As illustrated in Figure 5, the multi-scale feature fusion module comprises four parallel branches and a residual connection. Initially, we employ a convolutional operation with a kernel of size 1×1 to reduce the number of channels in the input feature map to 64. Following this, in the first branch, we sequentially execute a convolution with a kernel also of size 1×1, followed by another with a kernel of size 3×3. For the i-th(i∈{2,3,4}) branch of the module, the procedure commences with a convolution involving a kernel of size (2i−1)×1, proceeded by another convolution with a kernel of size 1×(2i−1). Finally, a dilated convolution operation with a kernel of size 3×3 and a dilation rate of 2i−1 is applied. This design strategy is aimed at extracting multi-scale information from the multi-modal fusion features, thereby enriching the representational power of the model. Next, the outputs from the four branches are stacked along the channel dimension, and the channel count of the stacked output is adjusted to match the input feature map’s channel count, using a convolution operation with a kernel size of 1×1. Finally, the adjusted result is connected to the input feature map via a residual connection. The entire fusion process can be described as follows:

(19) $$branch_{1}(x)={\mathrm{ConvBR}}_{3\times 3}({\mathrm{ConvBR}}_{1\times 1}(x))$$

(20) $$branch_{i}(x)={\mathrm{ConvBR}}_{3\times 3}^{2i-1}({\mathrm{ConvBR}}_{1\times (2i-1)}({\mathrm{ConvBR}}_{(2i-1)\times 1}({\mathrm{ConvBR}}_{1\times 1}(x)))),\;i\in \{2,3,4\}$$

(21) $$\mathrm{MSF}(x)={\mathrm{ConvBR}}_{1\times 1}(\mathrm{concat}(branch_{1}(x),branch_{2}(x),branch_{3}(x),branch_{4}(x)))+x$$

Herein, $branch_{i}(x)$ denotes the ith parallel branch, while x symbolizes the input feature map.

### 3.5. Loss Function

As depicted in Figure 1, at each stage of the decoder, the decoded output is upsampled to the size of the input image. Following this, a convolution operation with a single-channel convolution kernel of 1 × 1 is performed, and then a prediction saliency map is generated through a sigmoid activation function. The saliency maps predicted at each of the five stages are denoted as *O_i_* (*i* = 1, 2, ⋯, 5). Following the same process, we can also generate the predicted saliency map *O*^of^ corresponding to the output of the semantic localization module. This process can be described as follows:

(22) $$O_{i}=\mathrm{Sigmoid}({\mathrm{Conv}}_{1\times 1}({\mathrm{UP}}_{\mathrm{in}}(Decode_{i})))$$

(23) $$O^{\mathrm{of}}=\mathrm{Sigmoid}({\mathrm{Conv}}_{1\times 1}({\mathrm{UP}}_{\mathrm{in}}(F^{\mathrm{of}})))$$

Assuming the predicted saliency map is denoted as O, and the real saliency map is denoted as GT, the formula for calculating the loss value of the prediction results is as follows:

(24) Loss(O,GT)=Bce(O,GT)+Dice(O,GT)

(25) Bce(O,GT)=GT⋅logO+(1−GT)⋅log(1−O)

(26) $$Dice(O,GT)=1-\frac{2\cdot \mathrm{GT}\cdot O}{\left||GT||+||O|\right|}$$

Herein, Bce(⋅) represents the binary cross-entropy loss function, Dice(⋅) denotes the Dice loss function [43], and ||⋅|| represents the $L_{1}$ norm. The total loss function during the training phase is described as follows:

(27) $$L=\alpha \cdot \sum_{i=1}^{5}\mathrm{Loss}(O_{i},GT)+(1-\alpha )\cdot \mathrm{Loss}(O^{\mathrm{of}},\mathrm{GT})$$

wherein, α represents the weight coefficients. During the testing phase, *O*_1_ is the final prediction result of the model.

## 4. Experiments

Section 4.1 provides a detailed description of the implementation details, Section 4.2 discusses the sources of the datasets used, Section 4.3 introduces the setup of the evaluation metrics, Section 4.4 presents the comparison with the current state-of-the-art (SOTA) methods, and Section 4.5 is dedicated to the discussion of the ablation experiments. Together, these sections form the experimental analysis and evaluation part of the paper, comprehensively demonstrating the effectiveness and reliability of the research method.

### 4.1. Implementation Details

**Figure 6:**
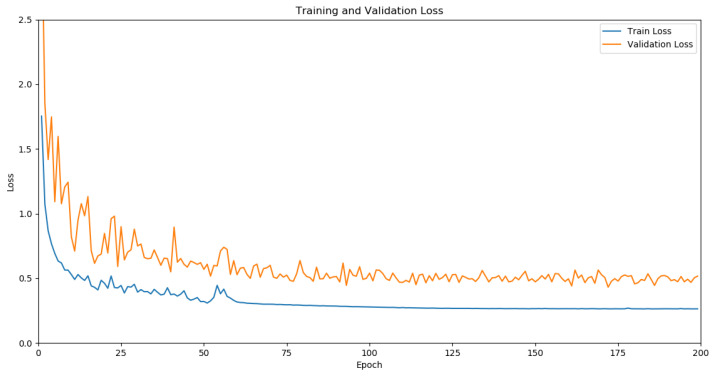
Training and validation loss curve.

The salient object detection method proposed in this paper is implemented based on the Pytorch framework [44,45], and all experimental procedures were carried out on a single NVIDIA RTX A6000 GPU(NVIDIA, Santa Clara City, CA, USA). The initialization parameters of the backbone model, ResNet50, are derived from a pre-trained model on ImageNet [46]. Specifically, both the RGB image branch and the depth image branch use a ResNet50 model for feature extraction, with the only difference being that the input channel number for the depth image branch is 1. To enhance the model’s generalization capability, various augmentation strategies, such as random flipping, rotation, and boundary cropping, were applied to all training images. Throughout the training process, the Adam optimizer was employed, with parameters set to β1 = 0.9 and β2 = 0.999, and a batch size of 10. The initial learning rate was set to 1 × 10^−4^ and was divided by 10 every 50 rounds. The dimensions of the input images were all adjusted to 768 × 768. The model converged within 200 rounds. In order to show the training process of our model more clearly, we report the training and validation loss curve of our network in Figure 6.

### 4.2. Datasets

In this study, SLMSF-Net was extensively evaluated across six widely used datasets, including NJU2K [47], NLPR [30], STERE [48], SSD [49], SIP [50], and DES [51]. These datasets contain 1985, 1000, 1000, 80, 929, and 135 images, respectively. For the training phase, we utilized 1485 images from the NJU2K dataset and 700 images from the NLPR dataset. During the testing phase, the remaining images from the NJU2K and NLPR datasets, as well as the entire STERE, SSD, SIP, and DES datasets were used.

### 4.3. Evaluation Metrics

We employed four widely used evaluation metrics to compare SLMSF-Net with previous state-of-the-art methods, namely E-Measure, F-measure, S-measure, and MAE.

E-Measure ($E_{\xi }$) is a saliency map evaluation method based on cognitive vision, capable of integrating statistical information at both the image level and local pixel level. This measurement strategy was proposed by [52] and is defined as follows:

(28) $$E_{\xi }=\frac{l}{W\times H}\sum_{i=1}^{W}\sum_{j=1}^{H}\xi (i,j)$$

Here, W and H represent the width and height of the saliency map, respectively, while ξ signifies the enhanced alignment matrix. E-measure has three different variants: maximum E-measure, adaptive E-measure, and average E-measure. In our experiments, we used the maximum E-measure (maxE) as the evaluation criterion.

F-measure ($F_{\beta }$) serves as a weighted harmonic mean of precision and recall. It is defined as follows:

(29) $$F_{\beta }=\frac{(1+\beta^{2})\mathrm{Precision}\times \mathrm{Recall}}{\beta^{2}\times \mathrm{Precision}+\mathrm{Recall}}$$

Here, β is a parameter used to balance Precision and Recall. In this study, we set β² to 0.3. Similar to E-measure, F-measure also has three different variants: maximum F-measure, adaptive F-measure, and average F-measure. In our experiments, we reported the results of the maximum F-measure (maxF).

S-measure ($S_{\alpha }$) is a method for evaluating structural similarity. It assesses from two perspectives: region awareness ($S_{r}$) and object awareness ($S_{o}$). It is defined as follows:

(30) $$S_{\alpha }=\alpha \times S_{o}+(1-\alpha )S_{r}$$

Here, α∈[0,1] is a hyperparameter used to balance between $S_{o}$ and $S_{r}$. In our experiments, α is set to 0.5.

MAE (Mean Absolute Error) represents the average per-pixel absolute error between the predicted saliency map S and the ground truth map GT. It is defined as follows:

(31) $$\mathrm{MAE}=\frac{1}{W\times H}\sum_{i=1}^{W}\sum_{j=1}^{H}\left|S(i,j)-GT(i,j)\right|$$

Here, W and H, respectively, denote the width and height of the saliency map. The MAE is normalized to a value in the [0, 1] interval.

### 4.4. Comparison with SOTA Methods

We compared the SLMSF-Net model proposed in this study with ten deep learning-based RGB-D saliency detection methods, including AFNet [53], HINet [54], C2DFNet [55], DCMF [56], CFIDNet [57], CIR-Net [58], DCF [59], DASNet [60], D3Net [50], and ICNet [61]. To ensure a fair comparison, we used the saliency maps provided by the authors. If the saliency maps were not provided, we computed them using the source code and model files provided by the authors.

#### 4.4.1. Quantitative Comparison

**Figure 7:**
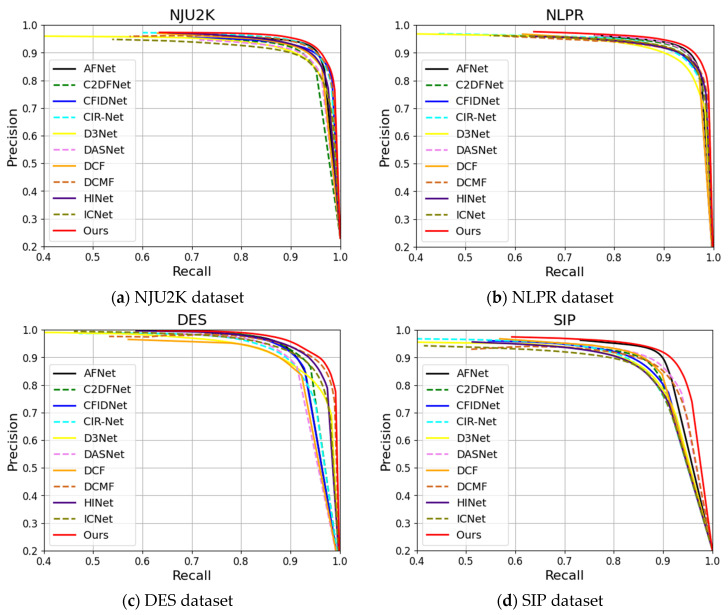

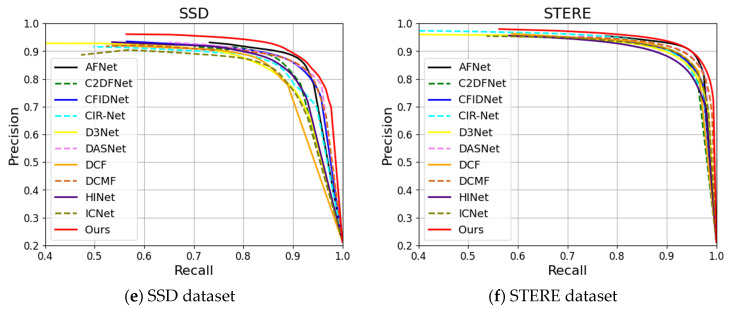
Comparison of precision-recall (P-R) curves for different methods across six RGB-D datasets. Our SLMSF-Net method is represented by a solid red line.

Figure 7 presents the comparison results of PR curves from different methods, while Table 1 presents the quantitative comparison results for four evaluation metrics. As shown in the figure and table, our PR curve outperforms all other comparison methods, whether on the NJU2K, NLPR, DES, SIP, SSD, or STERE datasets. This advantage is largely attributed to our designed semantic localization and multi-scale fusion strategies, which, respectively, achieve precise localization of salient objects and capture of detailed boundary information. Additionally, our designed depth attention module can effectively utilize depth information to enhance the model’s segmentation performance. Concurrently, the table data reflects the same conclusion, i.e., our method outperforms all comparison methods in performance on the NJU2K, NLPR, DES, SIP, SSD, and STERE datasets. Compared with the best comparison methods (AFNet, C2DFNet, and DCMF), we have improved the MAE, $\max F_{\beta }$, $\max E_{\xi }$, $S_{\alpha }$ evaluation metrics by 0.0002~0.0062, 0.2~1.8%, 0.09~1.46%, and 0.19~1.05%, respectively. Therefore, both the PR curves and evaluation metrics affirm the effectiveness and superiority of our method proposed for the RGB-D SOD task.

#### 4.4.2. Qualitative Comparison

**Figure 8:**
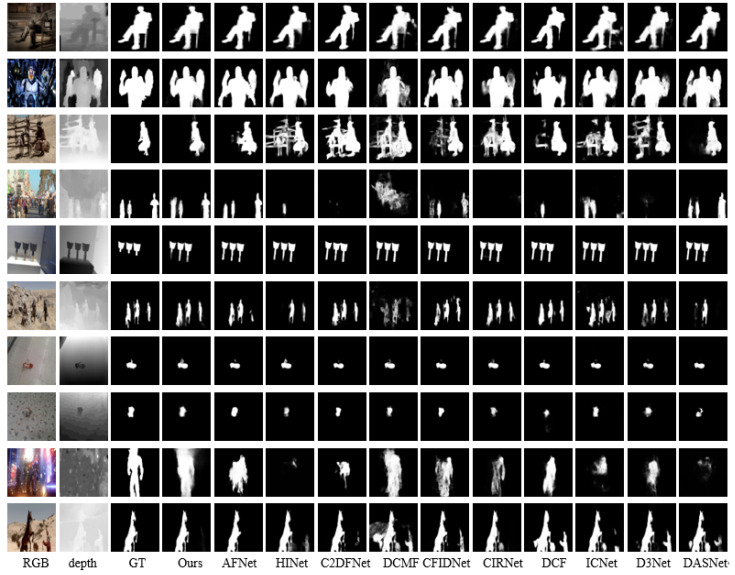
Visual comparison between SLMSF-Net and state-of-the-art RGB-D models.

For a qualitative comparison, we present a selection of representative visual examples in Figure 8. Upon observation, our method demonstrates superior performance in several challenging scenarios compared to other methods. Examples of these scenarios include situations where the foreground and background colors are similar (rows 1–2), in complex environments (rows 3–4), in scenes with multiple objects present (rows 5–6), for small object detection (rows 7–8), and under conditions of low-quality depth images (rows 9–10). These visual examples show that our method can more precisely locate salient objects and generate more accurate saliency maps.

### 4.5. Ablation Studies

As shown in Table 2, we conducted an in-depth ablation analysis to verify the effectiveness of each module. DAM represents the Deep Attention Module, SLM is the Semantic Localization Module, and MSFM stands for Multi-Scale Fusion Module. “Without DAM”, “without SLM”, and “without MSFM” refer to the models obtained after removing the DAM, SLM, and MSFM modules from the SLMSF-Net model, respectively. By comparing the data in the third column with the sixth column, we can clearly see that the introduction of the DAM module significantly improves the performance of the model. Similarly, by comparing the data in the fourth and sixth columns, we can see that the introduction of the SLM module can significantly enhance the performance of the model. Comparing the data in the fifth and sixth columns, we can see that adding the MSFM module will enhance the model’s performance. These results prove the importance of the three modules: the DAM module introduces depth image information, the SLM module realizes the precise semantic location of salient objects, and the MSFM module can fuse multi-scale features to refine the boundaries of salient objects. Each of these three functional modules resulted in a significant increase in model performance. In the last column, we can see that the SLMSF-Net model that incorporates these three modules achieved the best results.

## 5. Conclusions

In complex scenarios, achieving precise RGB-D salient object detection against multiple scales of objects and noisy backgrounds remains a daunting task. Current research primarily faces two major challenges: modality fusion and multi-level feature integration. To address these challenges, we propose an innovative RGB-D salient object detection network, the Semantic Localization and Multi-Scale Fusion Network (SLMSF-Net). This network comprises two main stages: encoding and decoding. In the encoding stage, SLMSF-Net utilizes ResNet50 to extract features from RGB and depth images and employs a depth attention module for the effective fusion of modal features. In the decoding stage, the network precisely locates salient objects through the semantic localization module and restores the detailed information of salient objects in the reverse decoder via the Multi-Scale Fusion Module. Rigorous experimental validation shows that SLMSF-Net exhibits superior accuracy and robustness on multiple RGB-D salient object detection datasets, outperforming existing technologies. In the future, we plan to further optimize the model, improve the attention mechanism, delve into refining edge details, and explore its application in RGB-T salient object detection tasks.

## Figures and Tables

**Table 1:** Comparison of results for four evaluation metrics—mean absolute error (MAE), maximum F-measure (maxF), maximum E-measure (maxE), and S-measure (S)—across six datasets. The symbol “↑” indicates that a higher value is better for the metric, while “↓” indicates that a lower value is better. The best performance in each row is highlighted in bold.

Datasets	Evaluation Metrics	Deep Learning-Based RGB-D Saliency Detection Methods										
		DASNet ICMM2020	D3Net TNNLS 2020	ICNet TIP2020	DCF CVPR2021	CIRNet TIP2022	CFIDNet NCA2022	DCMF TIP2022	C2DFNet TMM2022	HINet PR2023	AFNet NC2023	Ours
NJU2K [47]	MAE↓	0.0418	0.0467	0.0519	0.0357	0.0350	0.0378	0.0357	0.0387	0.0385	0.0317	**0.0315**
	maxF↑	0.9015	0.8993	0.8905	0.9147	0.9281	0.9148	0.9252	0.9089	0.9138	0.9282	**0.9352**
	maxE↑	0.9393	0.9381	0.9264	0.9504	0.9547	0.9464	0.9582	0.9425	0.9447	0.9578	**0.9615**
	S↑	0.9025	0.9	0.8941	0.9116	0.9252	0.9142	0.9247	0.9082	0.9153	0.9262	**0.9306**
NLPR [30]	MAE↓	0.0212	0.0298	0.0281	0.0217	0.0280	0.0256	0.0290	0.0217	0.0257	0.0201	**0.0199**
	maxF↑	0.9218	0.8968	0.9079	0.9118	0.9071	0.9054	0.9057	0.9166	0.9062	0.9249	**0.9298**
	maxE↑	0.9641	0.9529	0.9524	0.9628	0.9554	0.9553	0.9541	0.9605	0.9565	0.9684	**0.9693**
	S↑	0.9294	0.9118	0.9227	0.9239	0.9208	0.9219	0.9220	0.9279	0.9223	0.9362	**0.9388**
DES [51]	MAE↓	0.0246	0.0314	0.0266	0.0241	0.0287	0.0233	0.0232	0.0199	0.0215	0.0221	**0.0176**
	maxF↑	0.9025	0.8842	0.9132	0.8935	0.8917	0.9108	0.9239	0.9159	0.9220	0.9225	**0.9307**
	maxE↑	0.9390	0.9451	0.9598	0.9514	0.9407	0.9396	0.9679	0.9590	0.9670	0.9529	**0.9739**
	S↑	0.9047	0.8973	0.9201	0.9049	0.9067	0.9169	0.9324	0.9217	0.9274	0.9252	**0.9403**
SIP [50]	MAE↓	0.0508	0.0632	0.0695	0.0518	0.0685	0.0601	0.0623	0.0529	0.0656	0.0434	**0.0422**
	maxF↑	0.8864	0.861	0.8571	0.8844	0.8662	0.8699	0.8719	0.8770	0.8550	0.9089	**0.9114**
	maxE↑	0.9247	0.9085	0.9033	0.9217	0.9047	0.9088	0.9111	0.9160	0.8993	0.9389	**0.9408**
	S↑	0.8767	0.8603	0.8538	0.8756	0.8615	0.8638	0.8700	0.8715	0.8561	0.8959	**0.9045**
SSD [49]	MAE↓	0.0423	0.0585	0.0637	0.0498	0.0523	0.0504	0.0731	0.0478	0.0488	0.0383	**0.0321**
	maxF↑	0.8725	0.834	0.8414	0.8509	0.8547	0.8707	0.8108	0.8598	0.8524	0.8848	**0.9007**
	maxE↑	0.9298	0.9105	0.9025	0.9090	0.9119	0.9261	0.8970	0.9171	0.9160	0.9427	**0.9565**
	S↑	0.8846	0.8566	0.8484	0.8644	0.8725	0.8791	0.8382	0.8718	0.8652	0.8968	**0.9062**
STERE [48]	MAE↓	0.0368	0.0462	0.0446	0.0389	0.0457	0.0426	0.0433	0.0385	0.0490	0.0336	**0.0331**
	maxF↑	0.9043	0.8911	0.8978	0.9009	0.8966	0.8971	0.9061	0.8973	0.8828	0.9177	**0.9195**
	maxE↑	0.9436	0.9382	0.9415	0.9447	0.9388	0.9420	0.9463	0.9429	0.9325	0.9572	**0.9584**
	S↑	0.9104	0.8985	0.9025	0.9022	0.9013	0.9012	0.9097	0.9023	0.8919	0.9184	**0.9201**

**Table 2:** Comparison of ablation study results. The symbol “↑” indicates that a higher value is better for the metric, while “↓” indicates that a lower value is better. The best performance in each row is highlighted in bold.

Datasets	Evaluation Metrics	Without DAM	Without SLM	Without MSFM	SLMSF-Net
NJU2K [47]	MAE↓	0.0362	0.0352	0.0393	**0.0315**
	maxF↑	0.9214	0.9215	0.9165	**0.9352**
	maxE↑	0.9516	0.9508	0.9478	**0.9615**
	S↑	0.921	0.9225	0.9185	**0.9306**
NLPR [30]	MAE↓	0.0235	0.0234	0.0284	**0.0199**
	maxF↑	0.9226	0.9193	0.911	**0.9298**
	maxE↑	0.9627	0.964	0.9593	**0.9693**
	S↑	0.9328	0.9327	0.9238	**0.9388**
DES [51]	MAE↓	0.0191	0.0228	0.0231	**0.0176**
	maxF↑	0.9284	0.9174	0.9263	**0.9307**
	maxE↑	0.9704	0.9618	0.9687	**0.9739**
	S↑	0.9342	0.9260	0.9317	**0.9403**
SIP [50]	MAE↓	0.0569	0.0528	0.0600	**0.0422**
	maxF↑	0.8827	0.8916	0.8748	**0.9114**
	maxE↑	0.9154	0.9202	0.9119	**0.9408**
	S↑	0.8776	0.8830	0.8739	**0.9045**
SSD [49]	MAE↓	0.0537	0.0534	0.0548	**0.0321**
	maxF↑	0.8378	0.8381	0.8395	**0.9007**
	maxE↑	0.9093	0.9042	0.9045	**0.9565**
	S↑	0.8661	0.865	0.8658	**0.9062**
STERE [48]	MAE↓	0.0443	0.0376	0.0508	**0.0331**
	maxF↑	0.8919	0.9100	0.8906	**0.9195**
	maxE↑	0.9381	0.9479	0.9330	**0.9584**
	S↑	0.9014	0.9143	0.8986	**0.9201**

## Data Availability

Data are contained within the article.
